# Surgical Nuances to Reduce and Manage Cerebrospinal Fluid Leaks after Microvascular Decompression

**DOI:** 10.3390/jcm9040902

**Published:** 2020-03-25

**Authors:** Kyeong-O Go, Kihwan Hwang, Jung Ho Han

**Affiliations:** 1 Department of Neurosurgery, Gyeongsang National University Hospital, Gyengsangnam-do 52727, Korea; kko8353@gmail.com; 2 Department of Neurosurgery, Seoul National University Bundang Hospital, Gyeonggi-do 13620, Korea; coolghh@gmail.com; 3 Department of Neurosurgery, Seoul National University College of Medicine, Seoul 03080, Korea

**Keywords:** microvascular decompression, cerebrospinal fluid leakage, hemifacial spasm, trigeminal neuralgia, primary dural closure

## Abstract

Background: No dural substitute has proven to be complication-free in a large clinical trial, even suggesting some benefit during watertight closure. However, primary dural closure is not always possible due to dural shrinkage from electrocautery for dural bleeding. Objective: This study is performed to analyze the clinical outcomes related to cerebrospinal fluid (CSF) leakage after microvascular decompression (MVD) using a simple surgical technique. Methods: Three hundred and sixty consecutive cases were treated with MVD and followed up for more than one month after surgery. Bleeding from the cut veins during dural incision was controlled by pulling stay sutures instead of electrocautery to avoid dural shrinkage. Additionally, a wet cottonoid was placed on the cerebellar side dural flap to prevent dural dehydration. During dural closure, primary dural closure was always attempted. If not possible, a “plugging muscle” method was used for watertight dural closure. Results: The mean age was 54.1 ± 10.8 years (range, 24–85 years), and 238 (66.1%) were female. Primary MVD was performed in 345 (95.8%) patients. The mean operation time (from skin incision to skin closure) was 96.7 ± 33.0 min (range, 38–301 min). Primary dural closure was possible in 344 (95.6%) patients. The “plugging muscle method” was performed more frequently in patients older than 60 years (8 of 99 cases, 8.08%) than in younger cases (8 of 261 cases, 3.07%) (*p* = 0.039; chi-squared test). After surgery, 5 (1.4%) patients were treated for middle ear effusion, and another 5 (1.4%) patients experienced transient CSF rhinorrhea, which was spontaneously resolved within 1 to 7 days. No patients required additional treatments for CSF leakage. Conclusion: A simple technique using pulling stay sutures to stop bleeding from the dural edges and placing a wet cottonoid on the exposed dura can make primary dural closure easier.

## 1. Introduction

Cerebrospinal fluid (CSF) leakage is one of the most common complications after microvascular decompression (MVD) for neurovascular cross compression syndrome, including hemifacial spasm (HFS), trigeminal neuralgia, and glossopharyngeal neuralgia. Current treatments comprise reducing CSF pressure by continuous lumbar drainage or repeated spinal taps and administering antibiotics to prevent infections [1]. Failure of these treatments finally requires additional surgical intervention. Even after these treatments, fatal situations, such as pseudomeningocele, meningitis, and abscess formation, may complicate the postoperative course and lead to permanent deficits.

To prevent CSF leakage after MVD, it has been highly emphasized by Peter J. Jannetta to ensure watertight dural closure [2]. Additionally, primary dural closure (primary reapproximation and suturing of the dural edges) is the best seal without the introduction of autologous grafts of fat or artificial dural substitutes [3]. However, this is not always possible due to shrinkage of the dura mater only from exposure during surgery and/or electrocautery for dural bleeding.

Although many dural replacements have been introduced and used to ensure watertight dural closure, no substitute has proven to be complication-free in a large clinical trial, even suggesting some benefit [1,3]. Therefore, primary dural closure should be attempted during the closure of craniotomy or craniectomy for MVD.

In this study, clinical outcomes related to CSF leakage after retrosigmoid craniectomy with MVD were analyzed after using a simple technique to maintain the integrity of the dural flap, allowing for primary dural closure.

## 2. Materials and Methods

Between 2010 and January 2019, 360 consecutive cases were treated with retrosigmoid craniectomy, with MVD for HFS in 309 (85.8%) patients, trigeminal neuralgia in 50 (13.9%) patients, and glossopharyngeal neuralgia in one (0.3%) patient, and they were followed up more than one month after surgery. A retrospective review of medical records was performed to identify patients who experienced CSF leakage, including CSF rhinorrhea, otorrhea, pseudomeningocele, and/or incisional leak, during the initial hospital stay or by the first postoperative clinical follow-up usually at one month after surgery. The primary outcome was the primary dural closure rate using the surgical technique described below. Additionally, the author defined the secondary outcome as persistent CSF leakage that needs management with additional neurosurgical intervention such as continuous lumbar drainage, repeated spinal taps, and/or neurosurgical revision operation. Moreover, all patients with symptomatic CSF leakage were examed and assessed by otolaryngologists.

We collected all of the patient data based on information contained in hospital electronic medical records and followed the case record form, which was approved by the institutional review board. As a retrospective study, there was no risk to the subjects (minimum risk study), and the IRB committee approved (B-1903-528-105) the exemption of consent from the subjects.

### 2.1. Microvascular Decompression

**Figure 1:**
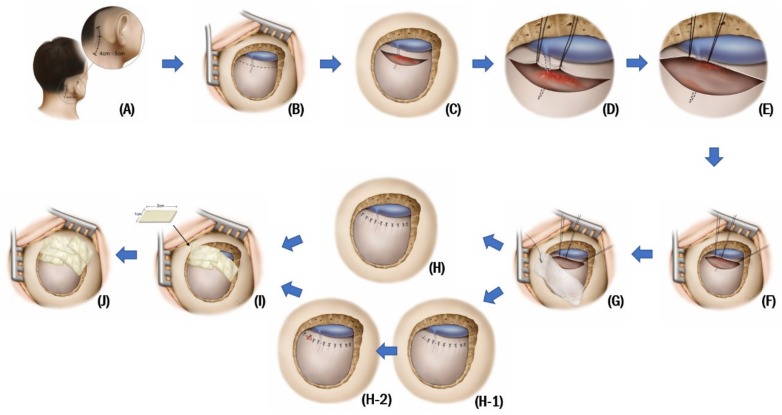
A 4- to 5-cm curvilinear skin incision was made along the hairline, and then retromastoid craniectomy was performed (**A**,**B**). A dural incision along the posterior margin of the sigmoid sinus was made approximately 2–3 mm away from the sinus margin for later dural repair (**C**). Upon bleeding from the cut dural veins, instead of electrocautery, one or two stay sutures were performed just around the cut vein. Thereafter, pulling these stay sutures tightly could easily cease bleeding from the cut veins (**D**,**E**). Stay sutures were performed along with the incised sinus-side dura mater as close as possible to the sinus margin and were pulled tightly to maximize visualization of an intracranial surgical corridor while minimizing cerebellar retraction (**F**). After completing the stay sutures, a wet cottonoid was placed on the cerebellar-side dura mater (**G**). During the closure, following the completion of the intradural procedures, primary dural closure was always attempted using interrupted sutures (**H**). If primary dural closure was not possible, a “plugging muscle” method, published elsewhere [5], was used (**H-1**,**H-2**). After dural closure, approximately 1 × 2 cm sized pieces of fibrinogen/thrombin-based collagen fleece (TachoComb^®^; Nycomed, Linz, Austria) were applied and overlapped in two or three layers on the dural incision site for additional sealing (**I**). After the bone edges of the mastoid air cells were thoroughly waxed, the bone edges were covered again using the remaining pieces of fibrinogen/thrombin-based collagen fleece (**J**).

All MVDs were performed by one neurosurgeon (JHH) and are described elsewhere [4]. With the patient in the supine position, the head was rotated approximately 20° to 30° away from the affected side without using head fixation. A 4- to 5-cm curvilinear skin incision was made along the hairline (Figure 1A), in which three quarters were below the mastoid notch in the cases of HFS and a half above the mastoid notch in cases of trigeminal neuralgia. Following the identification of the digastric groove after periosteal dissection, a 2–2.5 cm craniectomy was performed just below the digastric groove for HFS and was centered on the digastric groove for trigeminal neuralgia.

A dural incision along the posterior margin of the sigmoid sinus was made approximately 2–3 mm away from the sinus margin for later dural repair. Stay sutures were performed along with the incised sinus-side dura mater as close possible as to the sinus margin and were pulled tightly to maximize visualization of an intracranial surgical corridor while minimizing cerebellar retraction. During the dural incision, the dural veins were often cut, and they bled into the intracranial surgical corridor. At that time, instead of electrocautery, one or two stay sutures were performed just around the cut vein. Additionally, pulling these stay sutures tightly, as usual, could quickly cease bleeding from the cut veins (Figure 1B–E).

After completing the stay sutures (Figure 1F), a wet cottonoid was placed on the cerebellar-side dura mater, preventing the dura mater from becoming dehydrated by exposure to air and the light of the microscope during surgery (Figure 1G).

The offending vessel(s) were decompressed from the cranial nerves after the exploration of the whole intracranial portion of each involved cranial nerve, including the root exit/entry zone and cisternal segment.

After that main procedure, the operator washed out the surgical field by copious normal saline irrigation. It facilitates the elimination of almost all of the blood clots within the surgical corridor and inside the field. Through this process, the authors tried to prevent the increase in ICP that could be caused by secondary CSF dynamics change with residual blood components after surgery.

During the closure following the completion of the intradural procedures, primary dural closure was always attempted using interrupted sutures (Figure 1H). If primary dural closure was not possible, a “plugging muscle” method, published elsewhere and showing the lowest CSF leakage incidence of 0.29% after MVD [5], was used (Figure 1(H-1,H-2)). Provided the cut veins bled again after the release of the stay sutures, a stitch for dural closure close to the cut vein could control bleeding without electrocautery.

After dural closure, approximately 1 × 2-cm-sized pieces of fibrinogen/thrombin-based collagen fleece (TachoComb^®^; Nycomed, Linz, Austria), actually prepared to control bleeding from the dural sinuses during craniectomy, were applied and overlapped in two or three layers on the dural incision site for additional sealing with the yellow-coated part facing the dura (Figure 1I). After the bone edges of the mastoid air cells were thoroughly waxed, the bone edges were covered again using the remaining pieces of fibrinogen/thrombin-based collagen fleece (Figure 1J).

After that, in all of consecutive 360 patients, cranioplasty was performed, usually using polymethylmethacrylate (PMMA) bone cement. Finally, the deep and superficial muscles, fascia, and skin were approximated layer by layer.

Provided the postoperative course was uneventful, the patients were discharged between 48 and 72 h after surgery.

### 2.2. Statistical Analysis

A 2-sided *p*-value of 0.05 was regarded as statistically significant. Continuous variables were analyzed by Student’s *t*-test, and categorical variables were compared using the chi-squared test. Data analysis was performed using statistical software (IBM SPSS Statistics, Version 25 for Windows; IBM, Chicago, IL, USA).

## 3. Results

Among 360 cases treated with MVD, 238 (66.1%) were female. The mean age was 54.1 ± 10.8 years (range, 24–85 years). MVD for the right side was performed in 179 (49.7%) patients. Primary MVD was performed in 345 (95.8%) patients. Revision MVD was performed for 15 (4.2%) patients (2 referred from other institutes after primary MVD). The mean time for the entire decompression procedure (from skin incision to skin closure) was 96.7 ± 33.0 min (range, 38–301 min). The median and mode of the length of hospital stay after surgery was 3 days and 2 days, respectively.

Primary dural closure was possible in 344 (95.6%) cases. The “plugging muscle” method was required in 16 (4.4%) cases due to dural tearing or injury in the dural outer layer and/or the whole-layer defect when removing the inner table of the skull using a Kerrison punch or high-speed diamond drill, especially around the sigmoid sinus. The plugging muscle method was performed more frequently in patients older than 60 years (8 of 99 cases, 8.08%) than in younger cases (8 of 261 cases, 3.07%) (*p* = 0.039, chi-squared test). Female patients showed a lower primary dural closure rate (225 of 238 cases, 94.5%) than male patients (119 of 122 cases, 97.5%); however, the rate did not reach statistical significance (*p* = 0.191). Revision MVD and left or right directionality did not affect the primary dural closure rate.

After surgery, no patient exhibited a pseudomeningocele or incisional leak. Middle ear effusion (MEE) was confirmed in 5 (1.4%) patients with severe postoperative ear fullness. One of them who complaining of discomfort experienced myringotomy for the patient’s request and took oral antibiotics for two days. The remaining patients did not require any additional intervention for their MEE, which was resolved during observation.

Additionally, the other 5 (1.4%) patients exhibited transient watery rhinorrhea after surgery. One patient visited the emergency room with watery rhinorrhea at postoperative day (POD) 3, the day after discharge. Upon arrival, the watery rhinorrhea had already stopped and never recurred. Two patients described watery rhinorrhea at first clinical follow up after surgery that started at about POD 3 or 4 and lasted for approximately one week. They did not visit the hospital for evaluation of their rhinorrhea, and their watery rhinorrhea stopped spontaneously with a sense of pressure drop in their ears. One patient showed high fever and watery rhinorrhea at POD 2. Clinical and laboratory examinations, including CSF study, were done; however, there was no evidence of meningitis except atelectasis on chest X-ray. Watery rhinorrhea continued intermittently for four days and then stopped without any additional intervention. The last patient exhibited a small amount of watery rhinorrhea once at POD 3. The watery rhinorrhea never developed again. However, two days after discharge or POD 5, the patient visited the emergency room with a high fever and chills. Empirical intravenous antibiotics were used for one week to treat aseptic meningitis, which was revealed on CSF examination and culture study. Therefore, no patient displayed the secondary outcome, which is defined in this study as persistent CSF leakage such as CSF rhinorrhea, otorrhea, pseudomeningocele, and/or incisional leak managed with additional intervention.

## 4. Discussion

In this article, the author describes a straightforward and rapid technique to prevent CSF leakage that can occur after MVD through retrosigmoid craniectomy. To prevent CSF leakage, the surgeon should try various methods at each stage of surgery, but primary closure is fundamental. Besides, the author describes the surgical technique that enables primary dural closure in detail. After that, the extra fibrinogen/thrombin-based collagen fleece (TachoComb^®^; Nycomed, Linz, Austria) is applied to the dural incision site after use for hemostasis, and to the opening of the mastoid air cell, which enhances the watertight primary dural closure. Then, cranioplasty using artificial bone cement, which eliminates dead space on the craniectomy site, is performed. Finally, the soft tissues, from muscles to the skin, are closed watertight. With each step, the author could significantly reduce the occurrence of persistent CSF leakage that requires lumbar drainage or surgical intervention.

It is crucial to achieving tight and reliable dural closure while performing retromastoid craniectomy with MVD. The CSF leakage leads to increased morbidity, prolongation of hospital stay, and enhanced costs, as well as the need for revision surgery [6]. The previous reports have described techniques using autologous grafts, artificial dura, bone cement reconstruction, and postoperative lumbar drainage to address the problem of CSF leakage and these have been reported over the years with varying rates of success [5,7,8,9]. However, preparing these additional grafts intraoperatively and their postoperative management is time-consuming, increasing costs and causing patient inconvenience [10]. On the other hand, the bone cement reconstruction is a feasible way of preventing CSF leakage. Furthermore, continuous lumbar drainage can cause CSF over drainage, mechanical irritation, and is associated with the risk of meningitis, and these problems often require revision operations [11]. Therefore, it may be ideal to restore the opened dura mater to its original form to make it possible for watertight closure primarily.

Since the early days of neurosurgery, watertight dural closure has been advocated to avoid CSF leakage [1]. Primary dural closure (primary reapproximation and suturing of the dural edges) is the best seal without the introduction of autologous or artificial dural substitutes [3]. However, primary dural closure is not always possible due to the electrocautery of the dura mater and dehydration of the exposed dura mater.

First, the meningeal veins arise from the plexiform vessels in the dura mater. Additionally, they run the space between the inner and outer layers of the dura mater and drain into the dural sinuses. Thus, a dural incision along the margin of the sigmoid sinus often causes bleeding from the incised dural edges due to cutting off the meningeal veins draining to the sigmoid sinus. The electrocautery of the bleeding point of the dural edges results in shrinkage of the dura mater. Instead, pulling stay sutures tightly, just near the cut veins, typically to maximally visualize the surgical corridor, can compress the inner and outer layers of the dura mater. Compressing these two layers of the dura mater collapses the edge of the cut veins, easily stopping bleeding without the help of electrocautery. One report has suggested that it is necessary to shift the dural incision much closer to the venous sinuses to ensure primary dural closure [10]. However, this might cause profound sinus bleeding by the direct incision of the sinus wall when the sinus margin is not identified clearly, and by cutting the bridging point of the meningeal veins to the sinuses. Thus, dural incision 2-3 mm apart from the sinus margin would be safe.

A hemoclip can also be used instead of electrocautery for bleeding from the dural edges; however, removal of the used hemoclip after the intracranial procedure can result in another type of dural tearing, especially in cases of old age. In the elderly, the dura can be particularly fragile and adherent to the bone [1]. In the present study, the plugging muscle method was actually performed more frequently in patients older than 60 years because the outer layer of the dura was often peeled off with the inner table of the skull during removal of the skull using the Kerrison punch. Thus, the use of hemoclips might have an increased chance of dural tearing during removal, especially in these elderly patients. This tearing usually makes primary dural closure difficult. Additionally, high-speed diamond drilling can generate heat, which can also cause outer-layer dural injury or a dural defect. Thus, the dura should be protected cautiously from heating injury when using a high-speed diamond drill.

Second, the exposed dura, especially the cerebellar side, can dry up, which also makes primary dural closure difficult. A wet cottonoid placed on the exposed cerebellar side dura prevents the dura from being dehydrated by air and the light of the microscope [10]. Additionally, a wet cottonoid also prevents bleeding at the cerebellar-side dural edge from flowing into the surgical corridor and obstructing the surgeon’s field of vision. Additionally, the short operation time may reduce the possibility of dural dehydration and shrinkage by reducing the exposure time to the air and light of a microscope. For this, thorough preoperative planning will lead to early and accurate localization of the offending vessels [12].

Third, approximately 1 × 2-cm-sized pieces of fibrinogen/thrombin-based collagen fleece were applied on the dural incision site and bony edges in two or three layers in this study. In fact, the fleece was prepared for the control of bleeding from the dural sinuses during craniectomy. After surgery, the remaining pieces of the fleece were used for additional sealing of the dural closure site and bone edges. Recently, similar techniques have been introduced [6,13]. Even using the fleece for dural augmentation, CSF leakage still developed in their study, although the fleece showed some beneficial protective effect on the prevention of CSF leakage. These results indicated that dural augmentation using fleece alone is not sufficient to completely prevent CSF leakage.

Additionally, in one of the above studies, the fleece was used on the brain-side dura and skull side dura together [13]. However, intracranially placing the fleece material might cause a foreign body reaction, which may cause aseptic meningitis or adhesion between the cerebellar surface and dural mater. This adhesion would make it challenging to reexplore the surgical site in recurred cases. Based on the present study, the application of overlapping of the fleece in two or three layers on the skull side dura would be enough to prevent CSF leakage under the situation of primary closure.

Finally, even with this surgical technique, middle ear effusion and CSF rhinorrhea developed, although the CSF leakage defined in this case series did not occur. Although the events resolved spontaneously without additional intervention such as continuous lumbar drainage, repeated tapping, or surgical intervention, patients could develop these events that might be caused by irrigation fluid or CSF entering through the mastoid air cells during the procedure rather than the postoperative CSF leakage, considering that all the events stopped spontaneously. Thus, the opened mastoid air cells should be thoroughly sealed with bone wax as Dr. Jannetta emphasized the sealing of the opened mastoid air cells, or “wax the bone edges on the way in and on the way out” [2].

However, an issue that should be resolved concerns the timing of CSF leakage after MVD and additional intervention for CSF leakage. The case of CSF leakage that occurs after several years after MVD has been reported, but is very rare [14]. In general, it is known that CSF leakage after MVD is prevalent 1–2 weeks postoperatively [5,15]. Therefore, a one-month follow-up may be sufficient to evaluate the development of CSF leakage after MVD in the present study. In addition, several patients with 4-day or one-week lasting watery rhinorrhea were managed conservatively without any further intervention or were not managed ever for their rhinorrhea in this case series. These results might suggest the possibility of spontaneous healing of the CSF leakage site of the inflammatory reaction of the surgical site or fleece material. Therefore, in the case of suspected MEE or transient CSF rhinorrhea occurring within two weeks postoperatively, the authors suggest close observation of the clinical course rather than performing immediate lumbar drainage or surgical intervention. However, this policy may not be applicable for pseudomeningocele or incisional leakage, which did not develop in this case series, suggesting a continuous collection of CSF leakage in the surgical site.

The cranioplasty for bone defects is also crucial for preventing CSF leaks. As mentioned above, the CSF leakage was significantly lower in the group that filled bone defects using bone cement than titanium mesh [7,16,17,18]. However, fat graft-assisted titanium cranioplasty could also prevent postoperative CSF leak [17]. While the rigid titanium mesh maintains its contour after closing the scalp, the dead space between the dura suture site and mesh may also cause a driving force of negative pressure to make CSF leak at the dural closure site. In other words, the minimization of the dead spaces that inevitably occur even though filled with bone cement might facilitate the prevention of the CSF leakage. The authors filled the bone defects and the dead spaces with PMMA to restore the original shape of the contour as much as possible.

Besides the above, various factors in each step during MVD though retrosigmoid craniectomy, such as the sealing of opened mastoid air cells, deep muscle closure, and surgeons’ experiences and skills, are thought to contribute to preventing postoperative CSF leakage. Therefore, surgeons should perform all steps cautiously during surgical closure to prevent CSF leakage completely. Nonetheless, primary dural closure might be crucial as a first step in preventing CSF leakage.

## 5. Conclusions

A simple technique using pulling stay sutures to stop bleeding from the dural edges and placing a wet cottonoid on the exposed dura can make primary dural closure easier. The application of fibrinogen/thrombin-based collagen fleece on the dural closure site and bone edges, and elimination of dead space by bone cement cranioplasty might enhance positive effects on primary dural closure in terms of the prevention of CSF leakage after MVD.
